# Developmental validation of a novel multiple genotyping assay with 23 pigeon STR loci

**DOI:** 10.1093/fsr/owaf037

**Published:** 2025-10-28

**Authors:** Rongxing Wei, Jiaqian Le, Xueyuan Liu, Weian Du, Yangyang Zheng, Shiying Deng, Chengliang Yang, Weibin Wu, Rufeng Bai, Ling Chen

**Affiliations:** Guangzhou Key Laboratory of Forensic Multi-Omics for Precision Identification, School of Forensic Medicine, Southern Medical University, Guangzhou, China; Nanchang Police Dog Base of the Ministry of Public Security, Nanchang, China; Guangzhou Key Laboratory of Forensic Multi-Omics for Precision Identification, School of Forensic Medicine, Southern Medical University, Guangzhou, China; Zhaoqing Medical College, Zhaoqing, China; School of Medicine, Foshan University, Foshan, China; Department of Technology, Guangdong Homy Genetics Ltd, Foshan, China; Department of Technology, Guangdong Homy Genetics Ltd, Foshan, China; Guangzhou Key Laboratory of Forensic Multi-Omics for Precision Identification, School of Forensic Medicine, Southern Medical University, Guangzhou, China; Guangzhou Key Laboratory of Forensic Multi-Omics for Precision Identification, School of Forensic Medicine, Southern Medical University, Guangzhou, China; Criminal Justice College of China University of Political Science and Law, Beijing, China; Guangzhou Key Laboratory of Forensic Multi-Omics for Precision Identification, School of Forensic Medicine, Southern Medical University, Guangzhou, China

**Keywords:** forensic sciences, forensic genetics, developmental validation, STR, pigeon, individual identification, paternity test

## Abstract

Pigeons (*Columba livia*) occupy an important place in pigeon racing due to their extraordinary flying and nesting abilities. In order to maintain fairness in these races through accurate individual identification of pigeons using forensic techniques, we developed a novel multiple genotyping system containing 23 pigeon short tandem repeat (STR) polymorphisms and a chromo-helicase DNA binding gene (CHD). This system underwent rigorous validation in accordance with the guidelines set by the Scientific Working Group on DNA Analysis Methods, including species specificity, sensitivity, repeatability, reproducibility, size precision, and a population study of 219 unrelated individual pigeons. The results demonstrated that the novel pigeon STR system was specific, reproducible, accurate and consistent. Complete profiles were obtained with 62.5 pg of pigeon DNA input. Furthermore, the combined discrimination power of the 23 STR loci reached 1–3.666 4 × 10^−19^, while the combined exclusion power was 1–6.596 8 × 10^−5^. These findings underscore the high polymorphism and informativeness of pigeon STR genetic markers, making them ideal for pigeon individual identification and parentage testing.

## Introduction

Pigeons (*Columba livia*) are universally cherished across the globe [1, 2]. Thought to have been domesticated millennia ago, these birds are deeply intertwined with human activities and are primarily reared for culinary purposes, ornamental display, and competitive pigeon racing [1–3]. They have attracted the attention of countless enthusiasts and breeders worldwide [1, 2].

In ancient times, homing pigeons, renowned for their excellent spatial awareness and ability to navigate back from vast distances, served as vital long-distance communicators for humans [4]. In times of war, they were particularly instrumental in transmitting messages and directives. Nowadays, although they no longer deliver letters, homing pigeons continue to participate in numerous sports events and races held annually worldwide. These competitions have gained immense popularity across the globe [1]. Pigeon racing stands as a prevalent and lucrative sport that draws participants from all over the world. In some instances, racing pigeons can fetch prices amounting to millions of dollars [5–11]. Moreover, China is a prominent hub for pigeon racing, boasting over 1 300 pigeon organizations by the end of 2021, with more than 400 000 registered members in the China Pigeon Association, and over 20 000 races held annually [12].

The utilization of short tandem repeats (STRs) as molecular identifiers for animal discrimination and parentage verification has demonstrated remarkable efficacy across both routine and forensic applications [13–16]. Due to the substantial rewards offered in racing competition, ensuring the fairness and authenticity in the competition process is of utmost importance. Consequently, STR genotyping markers have been incorporated into the contests for the purpose of individual identification [1, 16–19].

The International Society for Animal Genetics (ISAG) currently recommends a core microsatellite panel comprising 16 STR loci (including *PIGN15*, *PIGN10*, *PIGN57*, *PIGN26*, *CliμD16*, *CliμD19*, *PIGN12*, *CliμD17*, *CliμT17*, *PIGN04*, *CliμD01*, *CliμD11*, *CliμD35*, *CliμT02*, *CliμT13*, *CliμT43*) for genetic analysis in domestic pigeons [16]. Previous studies have independently developed or applied various STR marker combinations. For instance, Lee et al*.* [20] developed a multiplex PCR system incorporating seven STR loci (*PG1*, *PG2*, *PG3*, *PG4*, *PG5*, *PG6*, *PG7*) and a chromo-helicase DNA- binding gene (*CHD*) marker for individual identification and parentage testing in racing pigeons; however, this system utilized a limited number of loci and suffered from insufficient sample size. Traxler et al. [21] employed seven microsatellite loci (*CliμD17*, *CliμT17*, *CliμD16*, *CliμD19*, *CliμD32*, *CliμT13*, *CliμD01*) for pedigree analysis within the Viennese Highflyer breed. Furthermore, Ramadan et al. [19] utilized eleven microsatellite markers (including *CliμD17*, *CliμT17*, *CliμD16*, *CliμD32*, *CliμT13*, *CliμD01*, *PG1*, *PG2*, *PG4*, *PG6*, and *PG7*) to analyze genetic diversity and relatedness across eleven Egyptian pigeon populations. Biała et al. [17] applied seven domestic pigeon microsatellite markers to investigate genetic variation and relationships among eight pure pigeon breeds and urban flocks. Podbielska et al. [1] evaluated the genetic structure of Polish racing pigeon populations based on the ISAG-recommended panel. Collectively, despite the availability of multiple STR analysis systems, limitations persist concerning multiplex polymerase chain reaction (PCR) compatibility, amplification stability, or individual discriminatory power. Therefore, building upon the ISAG-recommended loci, this study aims to further optimize the STR locus combination to construct a next-generation STR analysis panel with enhanced performance.

In this research, we developed a novel multiple genotyping system tailored for large-scale testing of pigeon genotype profiles. The system encompasses 23 STR loci and a *CHD* gene. The new system includes 23 STR markers, which cover all the 16 loci of the microsatellite panel recommended by the ISAG, including *PIGN15*, *PIGN10*, *PIGN57*, *PIGN26*, *CliμD16*, *CliμD19*, *PIGN12*, *CliμD17*, *CliμT17 (PG3)*, *PIGN04*, *CliμD01*, *CliμD11*, *CliμD35*, *CliμT02*, *CliμT13*, and *CliμT43* (*PG2*) [16]. Additionally, seven more STR loci have been incorporated, specifically *CliμD32*, *Cli12*, *Cli02*, *PG6*, *Cli06*, *PG5*, and *PG1* [19, 22]. A sex-determining marker, CHD, was added to determine the sex of the pigeons, which could be challenging to ascertain based on phenotypic characteristics, especially for nestlings [1]. To assess the utility of this system for individual identification and parentage testing of pigeons, a series of validation studies was conducted following the guidelines of the Scientific Working Group on DNA Analysis Methods (SWGDAM). This system boasts more innovative markers and heightened identification power, positioning it as a formidable tool for distinguishing individual pigeons and conducting paternity testing.

## Materials and methods

### Sample collection and DNA extraction

Feather samples were collected from 219 phenotypically diverse domestic pigeons (*C. livia*) originating from pigeon breeding farms in Foshan (Guangdong Province) and Taiyuan (Shanxi Province), China (Supplementary Table S5). To minimize close kinship, sampling was conducted under the guidance of experienced breeders, with individuals selected from different flocks, breeding lines, or geographic locations whenever possible, based on breeder records and phenotypic diversity. Due to confidentiality agreements with participating farms, detailed information on exact locations or familial relationships could not be disclosed. Each feather sample was gently plucked from the chest or tail region in the direction of natural feather growth to minimize tissue damage and ensure sufficient material for DNA extraction. Genomic DNA was extracted using a combined protocol involving the Chelex-100 method and the TIANamp Genomic DNA Kit (TIANGEN BIOTECH, Beijing, China). DNA concentration and purity were assessed using a spectrophotometer (e.g., NanoDrop) and confirmed by agarose gel electrophoresis.

### Primer preparation

The system utilized 16 ISAG-recommended STR markers for pigeon parentage and identification, alongside seven additional markers specifically selected to improve identification efficiency. For the 16 ISAG-recommended STR markers, some primer sequences were adopted directly, while others required redesign using Primer 5.0 to accommodate multiplex amplification with six fluorescent dyes. Additionally, primers for additional markers were manually designed with amplicon sizes ranging from 80 bp to 500 bp, adhering to the same parameters using Primer 5.0. The specificity of the primers was confirmed by BLAST analysis of the NCBI database. The primers for STRs were then tested by single-locus amplification to evaluate the amplification efficiency and specificity. The primers with good performance and without non-specific amplification were selected for the subsequent multiplex amplification. Primer selection was performed based on the following criteria: amplification efficiency between 90% and 110% (determined from standard curves); absence of non-specific amplification or primer-dimer formation; clear electropherogram peaks without stutter peaks or allelic dropout; and balanced signal intensities across all loci in multiplex PCR. The characteristics and primer sequences of the pigeon genetic markers are shown in Table 1.

**Table 1:** Information on pigeon genetic markers, repeat motif, primer sequences, fluorescent dye, size ranges, and primer concentration in the new system.

Locus	Repeat motif	Primer sequence (5′ → 3′)	Fluorescent dye	Length of amplified fragment (bp)	Concentration (μmol)
*CliμD32*	(CA)n	GTCTCACAGGAACAACTGCGCA	6-FAM	141–161	0.16
		TATAGCCAGTGCTTTTTTCTTGAG			0.16
*CliμD19*	(GT)n	GCGTTTGGATTTCTGGGA	6-FAM	168–176	0.15
		TCCGTGCATGGCAATTCT			0.15
*CliμD35*	(GT)n	TTCCCGGGAGCTTAAGGGATT	6-FAM	188–202	0.18
		CCAACTATCCTAGAGATTCTCCTC			0.18
*CliμT02*	(CATC)n	TTTCTCACAGTTTGTGTGTTAT	6-FAM	218–246	0.25
		AAGCAGCCTGCATGCTTCAGAG			0.25
*PIGN10*	(TATC)n	TAAACACTGATCATATAATTGTT	6-FAM	273–315	0.40
		AATGCTGGAAAGGTCTGACTTG			0.40
*PIGN26*	(ATTCT)n	TTCACCAAAGTCTGTGGACTC	6-FAM	370–470	0.35
		GATACTCAATGTGGGGGCGT			0.35
*PG6*	(AAAC)n	ATATTTTCCTATTACATACAA	HEX	144–156	0.15
		GAGGTGTAGTAACTGAAATAA			0.15
*CliμD11*	(CA)n	TAAGCACCTTCCTTCCTAAGC	HEX	174–196	0.20
		CTGACATTTGGATTTTAAAA			0.20
*Cli12*	(GT)n	ATTCAAATAGTGAGGCTTAG	HEX	230–254	0.40
		CCCACCTCTACAGACCCTGC			0.40
*PG2*	(ATGG)n	TCCAACCCACATTATTCTATG	HEX	274–306	0.15
		GATCTACCAGCCTAAGTGAAAC			0.15
*CHD*	W/Z	CCAAGGATGAGGAACTGTGCA	HEX	347–367	0.22
		TCTGCATCGCTAAATCCTTTA			0.22
*Cli06*	(GT)n	CAGAGGGTGAGTGGGAGCGA	TAMRA	144–152	0.24
		TGTAACTTTGAAAAACATGGA			0.24
*CliμD17*	(GT)n	GGTTCAGATAGAACAACTGCC	TAMRA	161–171	0.50
		CTAAGACATGCACATTTTTCAG			0.50
*PG3*	(CATC/GGAT)n	ATTGCTCAGGAAGGAGCAG	TAMRA	198–238	0.36
		CAAAAGGTAAGTTAAGGGC			0.36
*PG5*	(TTTG)n	AAACTTTCTGTACAGACCCACA	TAMRA	257–261	0.18
		TTCTACATCCCTGTTAAATGC			0.18
*PIGN04*	(TAGA)n	CCCTTCCAACTCAAACTATTCT	TAMRA	282–314	0.20
		AGATCTTTCCAGGGAGGGAAGG			0.20
*CliμD01*	(CA)n	TATTTATAAGGGGAATGTT	TAMRA	340–378	0.32
		GCAGTTACAGCATCAGCTGG			0.32
*PG1*	(TATC)n	GAGATGGTTTAAGGCAAATAA	ALEXA 568	129–149	0.40
		GCCTGTTAGTGGAAAATAGGTA			0.40
*Cli02*	(CA)n	ATGGCTTTATGCTCCCCCTTG	ALEXA 568	160–172	0.25
		CAAGGTACACTTTTAGGTAAG			0.25
*PIGN12*	(TCTCTA)n(ATCTAG)n	AGATCCAGCAGTCTTGAA	ALEXA 568	235–350	0.20
		CATCTAATGCGATAAATC			0.20
*PIGN15*	(GATA)n	AAGCTGTGGATGCACATTGTC	ALEXA 594	117–141	0.15
		ACCAGGCATTGGAGTCTT			0.15
*PIGN57*	(TAGA)n	ATTAGCTCACATTTCTGTGT	ALEXA 594	155–185	0.25
		TTATAAAAATGCACTATAAA			0.25
*CliμT13*	(TATC)n	TTCCCCATTAAAACATGCAA	ALEXA 594	221–241	0.30
		GTCTGTCGAGCAGTAACAGT			0.30
*CliμD16*	(GT)n	CAGTTATGGGAGAAGGTTCGAT	ALEXA 594	296–380	0.25
		AATGGGCTTTGGAAGTATAAAGT			0.25

### Multiple amplification system

The total PCR system was 10 μL, consisting of 2.0 μL primer mix, 4.0 μL of 2.5 × PCR Master Mix (containing 125 mmol/L Tris–HCl buffer, 125 mmol/L KCl, 7.5 mmol/L dNTPs, 5.0 mmol/L MgCl_2_), 0.5–2 ng DNA template, and ddH_2_O. The recommended cycling conditions are: initial denaturation at 95°C for 2 min, followed by 31 cycles of denaturation at 94°C for 30 s, annealing at 58°C for 1 min, extension at 72°C for 50 s, and a final extension at 68°C for 60 min.

### Electrophoresis and data analysis

For capillary electrophoresis, 1.0 μL of PCR product was mixed with 9.0 μL of deionized Hi-Di formamide and 0.5 μL of the Marker SIZ-500 size standard (AGCU ScienTech, Wuxi, China), and the amplified products were separated by capillary electrophoresis using an Applied Biosystems 3130xL Genetic Analyzer (Applied Biosystems, Waltham, MA, USA). Fragment sizes and genotyping were analyzed using GeneMapper ID-X software (Thermo Fisher Scientific, Waltham, MA, USA).

### Construction of allelic ladders

To create an allelic ladder for use in electrophoretic analysis, amplification products from all the different genotypes were cloned into the pMD18T vector and transformed into DH5α competent cells. The recombinant bacteria were selected and sent to Sangon Biotech (Shanghai, China) for sequencing to verify the structural size and base sequence of the inserted fragments. Once verified, recombinant plasmids containing different alleles were mixed as templates and stored at −20°C.

### Developmental validation

#### Species specificity

The species specificity of the multiplex amplification system was verified using DNA samples from human (9948), pig, cow, sheep, dog, goose, chicken, duck, mouse, cat, rabbit, fish, *Candida albicans*, and *Escherichia coli* (*E. coli*).

#### Sensitivity

Sensitivity verification was conducted using serial dilutions of control DNA template ranging from 2 ng to 15.625 pg (2 ng, 1 ng, 0.5 ng, 0.25 ng, 0.125 ng, and 62.5 pg, 31.25 pg, and 15.625 pg). Each sample was tested in triplicate.

#### Repeatability and reproducibility

From the population study, samples were randomly selected for amplification and electrophoretic separation by two different individuals using different electrophoresis instruments (Applied Biosystems 3500xL Genetic Analyzer and Applied Biosystems 3130xL Genetic Analyzer). Upon completion of the experiments, the data were compared to assess the repeatability and reproducibility of the results.

#### Size precision

The size precision and accuracy were assessed by collecting fragment size data from four complete injections of the allelic ladder on the Applied Biosystems 3130xL Genetic Analyzer. The average size of each allele was calculated and the standard deviation of the common allele at each locus was determined.

#### Population studies

Population genetics was investigated on a sample of 219 individuals using Power stats v1.2 (https://www.powerstats.de) and Arlequin 3.1 software (https://cmpg.unibe.ch/software/arlequin3/) to calculate allele frequencies, genetic diversity (GD), observed heterozygosity (Hobs), probability of exclusion (PE), polymorphic information content (PIC), power of discrimination (PD), typical paternity index (TPI), probability of matching (PM), and the *P*-values of Hardy–Weinberg equilibrium (HWE). The values of the combined power of discrimination (CPD) and the combined probability of exclusion (CPE) were computed with the following formula: CPD = 1 − ∏(1 − PD_i_), CPE = 1 − ∏(1 − PE_i_), where PD_i_ and PE_i_ represent the value of PD and PE of the i-th locus, respectively.

## Results and discussion

### Allelic ladders

**Figure 1:**
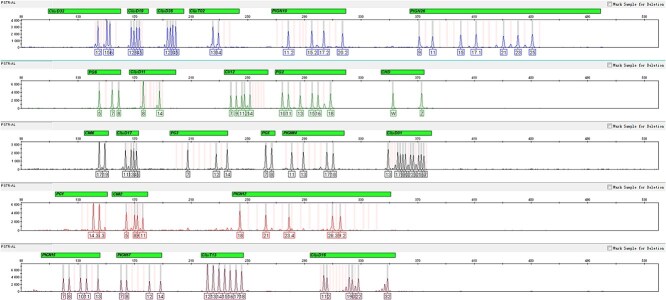
Allelic ladder of the new system, constructed with 23 short tandem repeat (STR) loci and chromo-helicase DNA binding gene (CHD).

The ladder for electrophoresis analysis was constructed by mixing products of single-plex PCR amplification of the recombinant plasmids. As depicted in Figure 1, all alleles from different loci exhibited distinct and characteristic peaks within their respective size ranges. Furthermore, the peak heights are balanced among different loci across all dye channels, suggesting a highly successful allelic ladder construction. Therefore, this standard allelic ladder can serve as a reliable reference for accurately identifying distinct amplification products.

### Species specificity

For the amplification, detection, and analysis of common animal DNA and microbial DNA, we found that only samples from chickens, ducks, and geese produced amplifiable peaks; all others (from other domestic animals, humans, and microbial DNA) failed. Specifically, chicken samples exhibited peaks at the CliμD32 locus, whereas both duck and goose samples showed peaks at both the CliμD32 and CHD loci.

CHD has been established as a common locus in certain non-ratite bird species and serves as a molecular marker for sex identification in these birds [23, 24]. Consequently, it produced peaks at corresponding positions in both ducks and geese. Based on this observation, we postulated that CliμD32 may also be a shared gene in non-ratite birds, which required further verification through the collection of additional bird samples in subsequent studies. Notably, the fragment length of the duck and goose amplification products at the CHD locus did not match that of the pigeons. Taken together, the system demonstrates good species specificity (Supplementary Figure S1).

It should be noted that cross-amplification was observed at the CliμD32 locus in non-target avian species, including chickens, ducks, and geese, which may reduce the forensic specificity of this individual marker. However, the full 23-locus STR profile is required for individual identification, and the presence of a single cross-reactive locus does not compromise the overall discriminatory power or specificity of the system when interpreted in combination with species-specific markers. For instance, the CHD locus, included for sex determination, showed no amplification in chickens and only weak amplification in ducks and geese—consistent with its high conservation among avian species. Importantly, CHD is not used for species identification but for determining genetic sex in pigeons. We emphasize that sample origin should be morphologically or genetically confirmed prior to analysis in forensic casework to avoid misidentification.

### Sensitivity study

**Figure 2:**
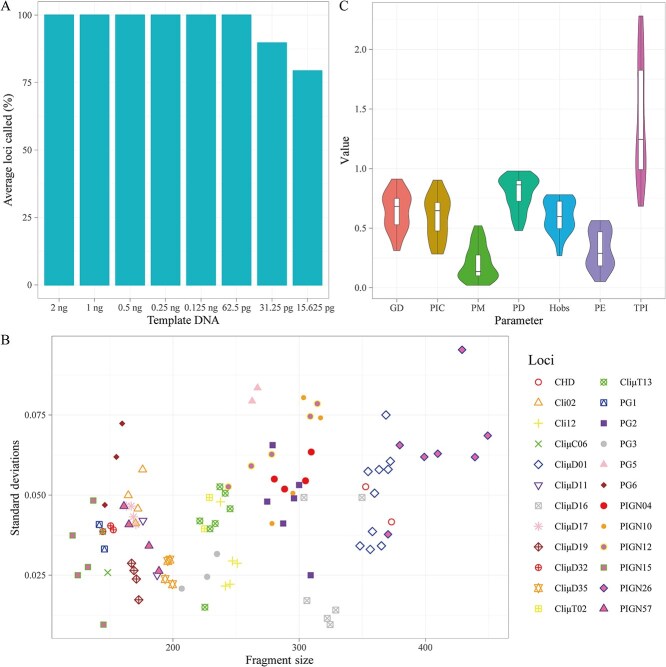
(A) The results of the sensitivity study based on the call rate. (B) The precision of the multiplex system across four injections on a 3130xL Genetic Analyzer. (C) Forensic parameters in the studied samples. GD: genetic diversity; PIC: polymorphism information content; PM: probability of matching; PD: power of discrimination; Hobs: observed heterozygosity; PE: probability of exclusion; TPI: typical paternity index.

To assess the minimum amount of DNA template required by the system, we performed sensitivity experiments. It was found that the peak height of the loci progressively decreased as the amount of DNA template diminished. When the DNA template quantity was 62.5 pg or more, all loci were successfully detected. However, at 31.25 pg of DNA template, the CliμD01 locus began to show peak loss, resulting in a locus detection rate of 89.66%. Furthermore, when the amount of DNA template was reduced to 15.625 pg, the locus detection rate dropped to 79.31%. Therefore, we recommend a minimum input DNA template quantity of 62.5 pg for optimal performance of this system (Figure 2A and Supplementary Figure S2). Feathers are widely recognized as a type of forensic material with insufficient nuclear DNA content, while 62.5 pg was identified as the minimum DNA input required to generate complete STR profiles under controlled conditions. For other sample types containing high-quality genomic DNA, such as tissue, blood, and body fluids, this amount may be sufficient; however, we agree that the recommended DNA input should be adjusted according to the sample type. Therefore, in practical applications, we recommend conducting preliminary experiments to optimize the DNA input and reaction conditions for specific sample types. For samples that are significantly degraded or known to be of low quality, we suggest using a higher starting amount and incorporating DNA repair enzymes or inhibitor removal steps to improve amplification efficiency.

### Repeatability and reproducibility

Repeatability testing was performed by two experimenters by repeating the system three times with control DNA using the same amplification conditions and instrumentation. For each test, the genotypes of all loci remained consistent. The samples were tested on two different instruments (3500xL and 3130xL). The results showed that the genotypes of all the samples were consistent across the two different instruments.

### Sizing precision

The precision of the allele sizes was calculated based on the four allele ladders analyzed. The standard deviation ranged from 0.010 bp (*CliμD16*) to 0.095 bp (*PIGN26*), with all values comfortably falling within the threshold of sizing precision (0.15 bp) specified in the Performance Check section of the 3130xL Genetic Analyzer User Guide. This indicated that the detection system had good precision (Figure 2B).

### Population study

The population study was comprehensively conducted by examining 219 individual pigeons using the newly developed system. The corresponding forensic parameters and allele frequencies are shown in Figure 2C and Supplementary Tables S1 and S2. The Hobs values ranged from 0.269 4 (*CliμD35*) to 0.780 8 (*PG2*). The GD values ranged from 0.311 8 (*CliμD35*) to 0.911 9 (*PIGN26*). The PM values ranged from 0.020 2 (*PIGN26*) to 0.520 2 (*CliμD35*). The PIC and TPI values ranged from 0.283 3 (*CliμD35*) to 0.903 0 (*PIGN26*) and 0.684 4 (*CliμD35*) to 2.281 3 (*PG2*), respectively. The CPD and CPE values for the 23 STR loci in pigeons were exceptionally high, at 1 − 3.666 4 × 10^−19^ and 1 − 6.596 8 × 10^−5^, respectively. These results underline the polymorphism and informativeness of the pigeon STR system, making it highly suitable for individual pigeon identification and parentage testing.

A parallel comparison was conducted between the STR panel developed in this study and the core loci panel recommended by the ISAG, using the same sample set (*n* = 219). The evaluation metrics included PIC, CPD, and CPE (Supplementary Tables S3 and S4). Preliminary results showed that the optimized panel in this study outperformed the conventional ISAG panel, with higher CPD (1 − 3.666 4 × 10^−19^  *vs.* 1 − 1.13 × 10^−13^) and CPE (1 − 6.596 8 × 10^−5^  *vs.* 1 − 1.98 × 10^−4^) (Table 2). Furthermore, we acknowledge that the ISAG-recommended loci have advantages in terms of international data sharing. Therefore, we propose that the panel developed in this study could serve as a complementary or upgraded alternative, particularly for applications requiring high-resolution individual identification and forensic analysis.

**Table 2:** Comparison of forensic efficiency parameters between the optimized short tandem repeat polymorphism panel developed in this study and the International Society for Animal Genetics (ISAG)-recommended core panel, based on the same sample set (*n* = 219).

Parameter	Optimized panel (this study)	ISAG-recommended panel
Mean PIC	0.591 1	0.627 9
CPD	1–3.666 4 × 10^−19^	1–1.13 × 10^−13^
CPE	1–6.596 8 × 10^−5^	1–1.98 × 10^−4^

PIC: polymorphic information content; CPD: combined power of discrimination; CPE: combined probability of exclusion.

We fully acknowledge that our samples of 219 pigeons from farms in China may not be fully representative of pigeon populations in other geographical regions (e.g., Europe or North America), due to potential differences in genetic backgrounds, farming practices, environmental conditions, and history of pathogen exposure. However, we believe our data remain highly valuable, as our sampling encompassed farms across several major pigeon breeding regions in China, such as the central, eastern, and southern parts, thereby capturing a notable degree of national diversity. Strengthening cooperative research with pigeon farms in western China represents a key direction for our future efforts.

Furthermore, regional breeding practices and selection pressures can lead to differences in allele frequencies. Therefore, we caution against the direct extrapolation of forensic statistics (e.g., match probabilities and exclusion power) derived from this study to populations outside of China without local validation. To enhance the generalizability of our conclusions, we recommend future studies to expand sampling across diverse geographical regions and ecological contexts, including urban, rural, and wild habitats, both within China and internationally. In addition, we advocate for the establishment of national or regional STR databases for pigeons to support standardized genetic monitoring, breed identification, and forensic applications. These efforts will be critical for validating the broad applicability of this STR panel in global contexts, including European, Middle Eastern, and American populations.

## Conclusion

Our research meticulously crafted a novel system with 23 pigeon STRs and a sex-determining CHD, covering all ISAG-recommended loci for microsatellite panels. The validation study has firmly established the system’s precision, specificity, reproducibility, stability, polymorphism, and informativeness in genotype determination. Population studies further underscore its effectiveness for individual identification and paternity testing. Thus, the system could be a tool for ensuring fairness in pigeon racing.

## Supplementary Material

Supplementary_Figure_owaf037

Supplementary_Table1-2_owaf037

Supplementary_Table3-4_owaf037

5_TFSR-2025-0021_Supplementary_Table_5_owaf037

## Notes

*Disclosure statement*: The authors declare that they have no conflicts of interest.

## References

[1] Podbielska A, Radko A. Genetic structure of racing pigeons (*Columba livia*) kept in Poland based on microsatellite markers. Genes (Basel). 2022;13:1175. 35885956 10.3390/genes13071175 PMC9318851

[2] Stringham SA, Mulroy EE, Xing J, et al. Divergence, convergence, and the ancestry of feral populations in the domestic rock pigeon. Curr Biol. 2012;22:302–308. 22264611 10.1016/j.cub.2011.12.045 PMC3288640

[3] Carlen E, Munshi-South J. Widespread genetic connectivity of feral pigeons across the Northeastern megacity. Evol Appl. 2021;14:150–162. 33519962 10.1111/eva.12972 PMC7819573

[4] Gugołek A, Jastrzębska A, Strychalski J. Wykorzystanie gołębi i innych gatunków ptaków w rekreacji człowieka. Wiad Zoot. 2016;54:90–95. Polish.

[5] Chang CC, Silva B, Huang HY, et al. Development and validation of KASP assays for the genotyping of racing performance-associated single nucleotide polymorphisms in pigeons. Genes (Basel). 2021;12:1383. 34573366 10.3390/genes12091383 PMC8468996

[6] Jędrzejczak-Silicka M, Yu Y, Cheng Y, et al. The influence of *LDHA* gene polymorphism on relative level of its expression in racing pigeons. Acta Scientiarum Polonorum Zootechnica. 2018;17:9–16.

[7] Jerolmack C . Animal archeology: domestic pigeons and the nature-culture dialectic. Qualitative Sociol Rev. 2007;3:74–95.

[8] Abolnik C . A current review of avian influenza in pigeons and doves (*Columbidae*). Vet Microbiol. 2014;170:181–196. 24667061 10.1016/j.vetmic.2014.02.042

[9] Proskura WS, Cichoń D, Grzesiak W, et al. Single nucleotide polymorphism in the *LDHA* gene as a potential marker for the racing performance of pigeons. J Poult Sci. 2014;51:364–368.

[10] Proskura WS, Dybus A, Łukaszewicz A, et al. The single nucleotide polymorphisms in lactate dehydrogenase-a (*LDHA*) and feather keratin (*F-KER*) genes and racing performance of domestic pigeon, Zesz Nauk. 2015;608:37–42.

[11] Ramadan S, Miyake T, Yamaura J, et al. *LDHA* gene is associated with pigeon survivability during racing competitions. PloS One. 2018;13:e195121. 10.1371/journal.pone.0195121 PMC5959059 29775483

[12] Wang JS . Racing pigeons from the “cloud” in Beijing: an annual event with a prize pool of millions, with 200,000 pigeons equipped with identity rings for tracking. Beijing (China): The Beijing News; 2022. Available from: https://www.bjnews.com.cn/detail/1667554554168081.html. Chinese.

[13] Budowle B, Garofano P, Hellman A, et al. Recommendations for animal DNA forensic and identity testing. Int J Leg Med. 2014;119:295–302. 10.1007/s00414-005-0545-9 15834735

[14] Linacre A, Gusmão L, Hecht W, et al. ISFG: recommendations regarding the use of non-human (animal) DNA in forensic genetic investigations. Forensic Sci Int Genet. 2011;5:501–505. 21106449 10.1016/j.fsigen.2010.10.017

[15] Iyengar A, Hadi S. Use of non-human DNA analysis in forensic science: a mini review. Med Sci Law. 2014;54:41–50. 23929675 10.1177/0025802413487522

[16] de Groot M, van Haeringen WA. An evaluation of the International Society for Animal Genetics recommended parentage and identification panel for the domestic pigeon (*Columba livia domestica*). Anim Genet. 2017;48:431–435. 28449233 10.1111/age.12555

[17] Biała A, Dybus A, Pawlina E, et al. Genetic diversity in eight pure breeds and urban form of domestic pigeon (*Columba livia var. domestica*) based on seven microsatellite loci. J Anim Plant Sci. 2015;25:1741–1745.

[18] Ando H, Kaneko S, Suzuki H, et al. Genetic diversity of the Japanese wood pigeon, Columba janthina, endemic to islands of East Asia, estimated by newly developed microsatellite markers. Zoolog Sci. 2011;28:891–896. 22132786 10.2108/zsj.28.891

[19] Ramadan S, Dawod A, El-Garhy O, et al. Genetic characterization of 11 microsatellite loci in Egyptian pigeons (*Columba livia domestica*) and their cross-species amplification in other Columbidae populations. Vet World. 2018;11:497–505. 29805216 10.14202/vetworld.2018.497-505 PMC5960790

[20] Lee JC, Tsai L, Kuan Y, et al. Racing pigeon identification using STR and chromo-helicase DNA binding gene markers. Electrophoresis. 2007;28:4274–4281. 18041042 10.1002/elps.200700063

[21] Traxler B, Brem G, Müller M, et al. Polymorphic DNA microsatellites in the domestic pigeon, *Columba Livia Var domestica*. Mol Ecol. 2000;9:366–368. 10736035 10.1046/j.1365-294x.2000.00874-2.x

[22] Zhang X, He Y, Zhang W, et al. Development of microsatellite marker system to determine the genetic diversity of experimental chicken, duck, goose, and pigeon populations. Biomed Res Int. 2021;2021:8851888. 33511214 10.1155/2021/8851888 PMC7822670

[23] Vucicevic M, Stevanov-Pavlovic M, Stevanovic J, et al. Sex determination in 58 bird species and evaluation of *CHD* gene as a universal molecular marker in bird sexing. Zoo Biol. 2013;32:269–276. 22553188 10.1002/zoo.21010

[24] Griffiths R, Double MC, Orr K, et al. A DNA test to sex most birds. Mol Ecol. 1998;7:1071–1075. 9711866 10.1046/j.1365-294x.1998.00389.x

