# Supplementary figures and images for: Developmental validation of a novel multiple genotyping assay with 23 pigeon STR loci
Source: Forensic Sci Res. 2025 Oct 28;10(4):owaf037. doi: 10.1093/fsr/owaf037 (PMC12724078; doi:10.1093/fsr/owaf037)

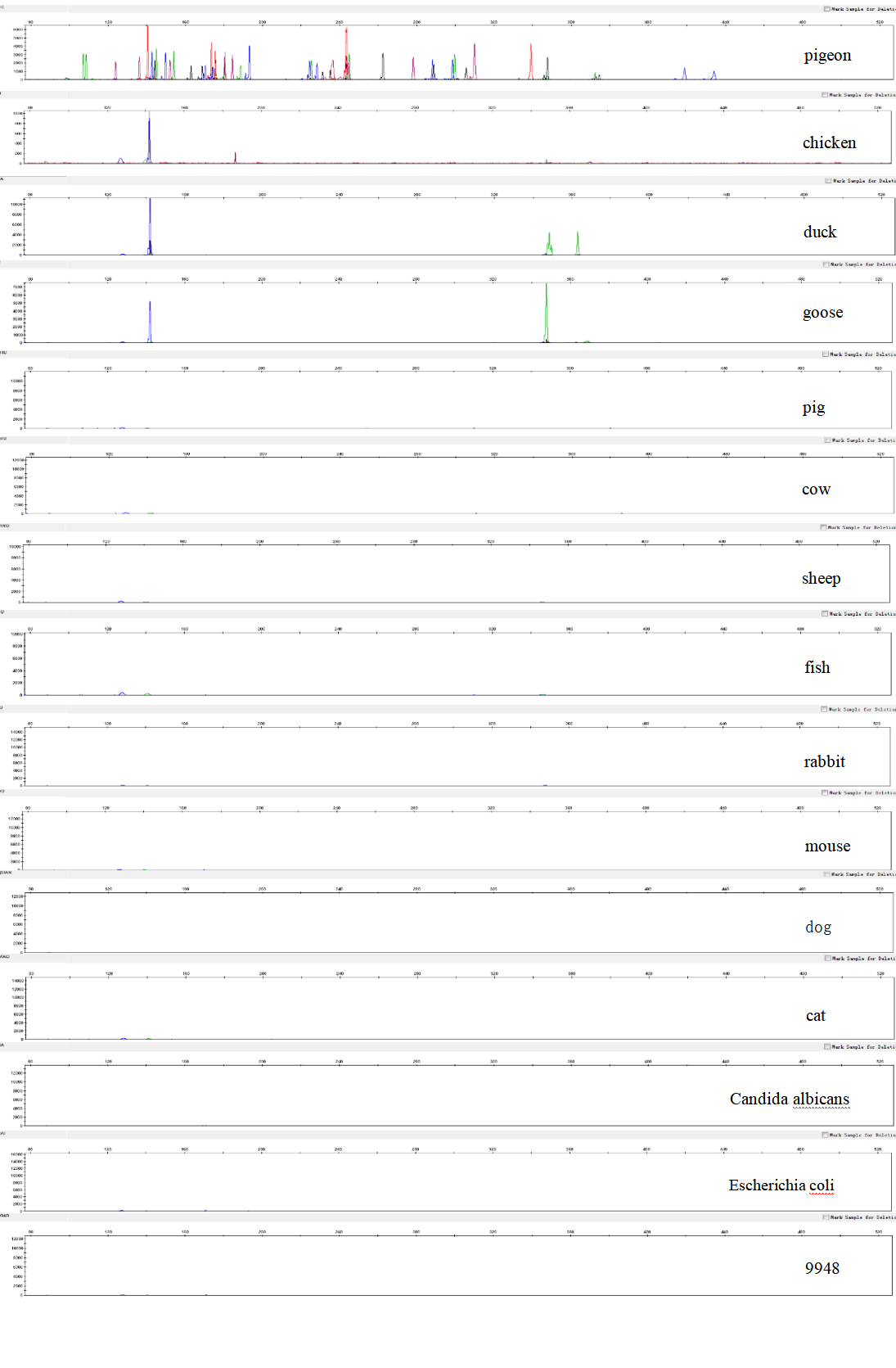


Fig. S1. The result of species specific study.


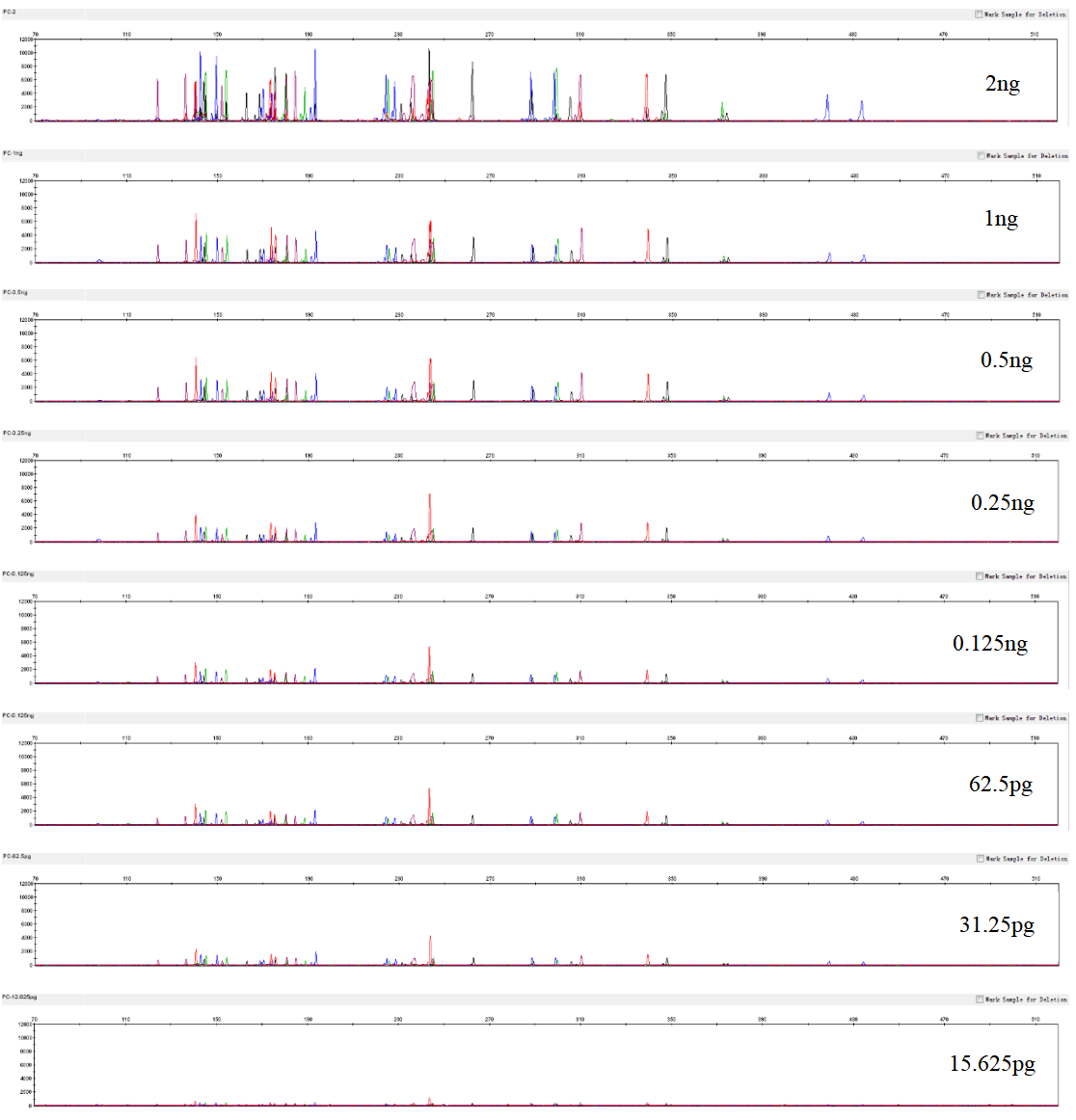


Fig. S2. Peak height results of the sensitivity study.

Supplement: Supplementary_Figure_owaf037 [file supplementary_figure_owaf037.docx]
